# Supplementary material for: Exploring nomophobia with a German adaption of the nomophobia questionnaire (NMP-Q-D)
Source: PLoS One. 2022 Dec 30;17(12):e0279379. doi: 10.1371/journal.pone.0279379 (PMC9803150; doi:10.1371/journal.pone.0279379)
Supplement: Attachment — Submitted filename: Response_to_Reviewer_nomophobia_7_10_22.pdf [file pone.0279379.s003.pdf]

PFH | PRIVATE UNIVERSITY of Applied Sciences

Prof. Dr. Yvonne Görlich
Psychological diagnostics and differential psychology
PFH Private University of Applied Sciences
Weender Landstraße 3-7
37073 Göttingen, Germany
Tel.: ++49 551 547 00 442
Email: goerlich@pfh.de

Göttingen, October 7, 20222

Dear Prof. Arpaci,
Thank you very much for the evaluation of our manuscript and the valuable input for improvements. We have prepared a revised version accordingly. A list of changes and answers to all points raised follow on the next pages. The changes in the manuscript are highlighted in yellow.

Best regards

Yvonne Görlich

Changes to the manuscript:

- A new table 3 was added with the fit statistics for the confirmatory factor analysis. Three new figures illustrate the details. This is referred to in the text.
- In tables 6 and 7, CR, AVE and square roots of AVE were added for all scales. This is referred to in the text.
- In the discussion, the point was added that this was a cross-sectional study and that a possible common method bias was checked factor-analytically via Harman's single factor score.

PFH | PRIVATE UNIVERSITY of Applied Sciences

Answers to the Reviewers' queries

(for clarity, I repeat the Referees' feedback in blue in front of each of my answers)

Reviewer #1: Manuscript Number: PONE-D-22-09552

Comments to the Author

1. Is the manuscript technically sound, and do the data support the conclusions?

The manuscript must describe a technically sound piece of scientific research with data that supports the conclusions. Experiments must have been conducted rigorously, with appropriate controls, replication, and sample sizes. The conclusions must be drawn appropriately based on the data presented.

Reviewer #1: Yes

---

2. Has the statistical analysis been performed appropriately and rigorously?

Reviewer #1: Yes

---

3. Have the authors made all data underlying the findings in their manuscript fully available?

The PLOS Data policy requires authors to make all data underlying the findings described in their manuscript fully available without restriction, with rare exception (please refer to the Data Availability Statement in the manuscript PDF file). The data should be provided as part of the manuscript or its supporting information, or deposited to a public repository. For example, in addition to summary statistics, the data points behind means, medians and variance measures should be available. If there are restrictions on publicly sharing data—e.g. participant privacy or use of data from a third party—those must be specified.

Reviewer #1: No

Data are now available in the supplements.

---

4. Is the manuscript presented in an intelligible fashion and written in standard English?

PLOS ONE does not copyedit accepted manuscripts, so the language in submitted articles must be clear, correct, and unambiguous. Any typographical or grammatical errors should be corrected at revision, so please note any specific errors here.

Reviewer #1: Yes

---

5. Review Comments to the Author

Please use the space provided to explain your answers to the questions above. You may also include additional comments for the author, including concerns about dual publication, research ethics, or publication ethics. (Please upload your review as an attachment if it exceeds 20,000 characters)

Reviewer #1: Manuscript Number: PONE-D-22-09552
Article Type: Research Article
Full Title: Exploring Nomophobia with a German adaption of the Nomophobia Questionnaire (NMP-Q-D)
Objective: The present study aimed to translate the nomophobia questionnaire (NMP-Q) into German, validate NMP-Q-D, and use it for exploring nomophobia in relation to fear of missing out, smartphone addiction, depression, anxiety, stress, psychological well-being, satisfaction with life, and big five personality traits (i.e., extraversion, neuroticism, openness to experience, conscientiousness, and agreeableness).
The manuscript needs more work and effort in each section:
1. Since this is a cross-sectional study and applied several scales at the same time common method bias may arise from certain tendencies that respondents apply. Therefore, common method bias should be an important concern and assessed.

In the discussion, the point was made that this was a cross-sectional study and a possible common method bias was checked factor-analytically via Harman's single factor score.

2. The instrument is formed by diverse demographic variables along with seven different scales including NMP-Q-D, FoMO, SAS-SV, BFI-10, DASS-21, WHO-5, and SWLS. Since this was a scale adaptation study:
a. Convergent and discriminant validity should be tested.

Was checked via the proposed indices CR, AVE and square roots of AVE.

b. CR and AVE values should also be reported in the matrix to test convergent and discriminant validity.

In tables 6 and 7, CR and AVE were added for all scales.

c. Square root of the AVE (show in the diagonals) and AVE values should also be reported in the correlation matrix table.

In tables 6 and 7, the square roots of AVE are now shown in the diagonals for all scales.

d. Model fit estimates including GFI, AGFI, and SRMR should be reported.

A new table 3 was added, showing the fit statistics for the confirmatory factor analysis (including GFI, AGFI, and SRMR).

3. Reliability and validity results of the used scales (i.e., FoMO, SAS-SV, BFI-10, DASS-21, WHO-5, and SWLS) should be reported.

In tables 6 and 7, CR, AVE and the square roots of the AVE are also given for all scales used. This shows the reliability and validity of the scales.

4. For confirmatory factor analysis tree models were tested: A unidimensional model (model 1), an uncorrelated four-factor model (model 2) and a hierarchical model with one second-order factor explaining the four nomophobia factors (model 3). Provide a figure to visualize the models, at least the model with the best model fit. Figures should be in high resolution (TIFF images with minimum 300 dpi).

For each tested model, a figure is now provided (figure 1 to figure 3).

5. Table 7 shows the results of the multiple regression analysis at measure time 1. In the table, Std. Deviation can be Std. Error, please check.

Thank you for pointing this out, it should indeed read “Std. Error” and has been changed accordingly.
