# Supplementary material for: Exploring nomophobia with a German adaption of the nomophobia questionnaire (NMP-Q-D)
Source: PLoS One. 2022 Dec 30;17(12):e0279379. doi: 10.1371/journal.pone.0279379 (PMC9803150; doi:10.1371/journal.pone.0279379)
Supplement: Attachment — Submitted filename: Response_to_Reviewer_nomophobia_29_11_22.pdf [file pone.0279379.s004.pdf]

PFH | PRIVATE UNIVERSITY of Applied Sciences

Prof. Dr. Yvonne Görlich
Psychological diagnostics and differential psychology
PFH Private University of Applied Sciences
Weender Landstraße 3-7
37073 Göttingen, Germany
Tel.: ++49 551 547 00 442
Email: goerlich@pfh.de

Göttingen, November 29, 2022

Dear Prof. Arpaci,

Thank you very much for the evaluation of our manuscript and the valuable input for improvements. We have prepared a revised version accordingly. The answers to all points raised can be found below. The changes in the manuscript are highlighted in yellow.

Best regards

Yvonne Görlich

PFH | PRIVATE UNIVERSITY of Applied Sciences

**Answers to journal requirements and editor comments**

(for clarity, I repeat the Referees' feedback in blue in front of each of my answers)

Journal Requirements:

Please review your reference list to ensure that it is complete and correct. If you have cited papers that have been retracted, please include the rationale for doing so in the manuscript text, or remove these references and replace them with relevant current references. Any changes to the reference list should be mentioned in the rebuttal letter that accompanies your revised manuscript. If you need to cite a retracted article, indicate the article's retracted status in the References list and also include a citation and full reference for the retraction notice.

We reviewed the reference list and found no issues.

Additional Editor Comments (if provided):

There are still some minor issues that need to be fixed. I kindly ask the authors to proofread the manuscript. There are (much too) many instances of using the typos, wrong tense and voice, throughout the manuscript. For example, in Table 2 "Kommunalitäten" will be "Communalities." In Table 6, "two-sided" will be "two-tailed." Again, in Table 6, significance level of the correlation coefficients should be re-checked. For example, the correlation coefficient (r) between Extraversion and Age is reported as r= .095 **p < .01. But correlation coefficient (r) should be over 10 for a significance level of 90% (r >.10, p < .01).

We proofread the manuscript, corrected multiple issues (e.g. tense, voice, communalities, two-tailed), and marked all corrections.

We re-calculated the correlation scores and checked the significance levels and found no discrepancies. We added the following sentence in the discussion: "The fact that even small correlations (e.g. NMP-Q-D and age correlated with r=-.094) became significant in sample 1 is due to the relatively large sample size."
